# The association between lifestyles and health conditions and the choice of traditional Chinese medical treatment in China: A latent class analysis

**DOI:** 10.1097/MD.0000000000032422

**Published:** 2022-12-23

**Authors:** Xueping Ma, Mohan Wang, Juan Ma, Zhengjun Zhang, Yu Hao, Ning Yan

**Affiliations:** a Heart Centre & Department of Cardiovascular Diseases, General Hospital of Ningxia Medical University, Yinchuan, Ningxia, China; b Ningxia Key Laboratory of Vascular Injury and Repair Research, Ningxia Medical University, Yinchuan, Ningxia, China; c Clinical Medical College, Ningxia Medical University, Yinchuan, Ningxia, China.

**Keywords:** Chinese Family Panel Studies (CFPS), health conditions, latent class analysis (LCA), lifestyles, traditional Chinese medicine (TCM)

## Abstract

Traditional Chinese medicine (TCM) plays a major role in preventing and treating the disease, however, it is also facing a slice of challenges as fewer choices of TCM treatment. Although lifestyles and health conditions might be paramount influencing factors for the choice of TCM treatment, the relative evidence is scarce. The current observational study was designed to evaluate this association.

A total of 24,173 Chinese individuals with a mean age of 47.3 years from the Chinese Family Panel Studies 2014 were selected. The choice of TCM treatment was acquired by the self-report questionnaire. Latent class analysis was employed to identify clusters of lifestyles and health conditions. The binary logistic regression model was employed to examine the association between lifestyles, health conditions and the choice of TCM treatment.

Lifestyles and health conditions were classified into 3 classes with latent class analysis, healthy group, unhealthy behavior group, and physical inactivity group. After controlling for potential confounding factors, the results showed individuals in unhealthy behavior group (odds ratio = 1.51, 95% confidence interval: 1.35–1.68, *P* < .001) or physical inactivity group (odds ratio = 1.11, 95% confidence interval: 1.02–1.22, *P* = .019) were more likely to visit TCM doctors than healthy group. Sex-specific difference was observed, the relationship still existed among the males.

The current study revealed the relationship between lifestyles, health conditions and the choice of TCM treatment. This will provide evidence for the TCM development and provide support for further research.

## 1. Introduction

Traditional medicine, has a long history, is the sum total of the knowledge, skill, and practices based on the theories, beliefs, and experiences indigenous to different cultures, whether explicable or not, used in the maintenance of health as well as in the prevention, diagnosis, improvement or treatment of physical and mental illness.^[1]^ Traditional Chinese medicine (TCM) has a >2500-year history and it is an integral part of Chinese culture.^[2]^ TCM runs through the entire process of prevention and treatment of chronic diseases, with both preventive and curative effects.^[3]^ As a popular form of complementary and alternative medicine, TCM is a fully institutionalized part of Chinese healthcare and widely used with Western medicine.^[4]^ In 2006, the TCM sector provided care for over 200 million outpatients and 7 million inpatients, accounting for about one fifth of health care in China.^[5]^ In the past several decades, rate of TCM use in developed countries has been on the rise,^[6]^ as an example, for Australia and South Korea are 69% and 75%, respectively.^[7]^ In addition, among many developing countries, 40% in China and Colombia, 71% in Chile, and up to 80% in quite a few African countries.^[8]^ Besides, the World Health Organization Traditional Medicine Strategy 2014 to 2023 has specified the goal of promoting universal health coverage by integrating traditional and complementary medicine services into health care service delivery and self-health care.^[9]^

Popular use of TCM and increasing consumer demand has been accompanied by a growth in research, and an increase in evidence-based approaches.^[7]^ In the history of the development of TCM in recent century, TCM has experienced glory and confusion.^[10]^ Although the development of TCM has improved in last few years, opportunities always coexist with challenges.^[10]^ In few past decades, under the influence of scientism, coupled with the fierce impact of Western medicine, TCM has experienced setbacks. In the current era, in the process of severe acute respiratory syndrome and coronavirus disease 2019 virus prevention and treatment, the curative effect of TCM is recognized by the World Health Organization.^[10,11]^ Although TCM has played an increasingly important role in disease prevention and treatment, TCM has been marginalized in the healthcare system and most of the Western-trained doctors are not willing to recommend TCM to patients.^[12]^ A previous study investigated outpatients among general hospital and Chinese medicine hospital, the results revealed about 38% chose TCM service.^[13]^ It was reported that among the 928 Chinese residents, 15.4% chose TCM first when seeking medical treatment, and 64.5% of those visited TCM doctor ever.^[14]^ A study among 945 old adults manifested approximately 50.11% of the elderly have had TCM visits in the past year.^[15]^ However, there are few studies on the influencing factors about the choice of TCM.

Traditional medicine is essential for diverse medical treatments and can develop the advantages of Chinese medicine further, but it has not been officially recognized by most people.^[16]^ Thus, knowing the information on influencing factors of choosing TCM treatment would potentially benefit the further application of TCM and awareness of the influencing factors of TCM treatment choice to policy-makers is, therefore, paramount. Lifestyles and health conditions, such as smoking, drinking, exercise, chronic disease, etc, might be paramount influencing factors for the choice of TCM treatment, but there are lack of corresponding evidence and research. Thus, in current study, we mainly aimed to explore the association between lifestyles and health condition and the choice of TCM using latent class analysis (LCA) in a large Chinese sample.

## 2. Materials and Methods

### 2.1. Study sample

The data used in this study came from the survey of the Chinese Family Panel Studies (CFPS), launched by 985 Program of Peking University and carried out by the Institute of Social Science Survey of Peking University, which is a nearly nationwide, comprehensive, longitudinal social survey.^[13]^ The CFPS covers 25 provinces and represents 94.5% of the total population in mainland China. A probability proportionate to size sampling method was developed to recruit the participants. A similar sampling procedure was described previously.^[17]^ The CFPS is designed to collect individual-, family- and community-level longitudinal data.^[18]^ In 2014 survey year, due to the TCM service information were collected and considered in current study, after excluding the samples with severe missing variables and a final valid sample of 24,173 respondents were included in analysis. Subjects were included if they had complete data related to TCM service choice; and age, gender, marital status, residency, smoking, drinking, exercise, income level, social status, chronic disease, self-rated health data at baseline. This survey has been ethically reviewed, and provides real and reliable data for academic research and national and social policy decisions.

### 2.2. Dependent variable

Traditional Chinese medical treatment choice was obtained by questions: “Would you choose traditional Chinese medicine doctor?.” Responses are contained in 3 options, 1 = yes, 2 = no, and 3 = doesn’t matter (few subjects chose 3 in this study). And then categorized as yes (response = 1) or no (response = 2 or 3).

### 2.3. Covariates

Independent variables as age, gender, educational level, marital status, residency, ever worked, income level, social status, and life satisfaction. Age (continuous variable). Sex was categorized as male or female. Education level was categorized as illiterate, primary school, junior high school, senior high school and above. Marital status was categorized as married, cohabitating, divorced, separated, widowed, or never married. Residency was categorized as rural or urban. Income level was measured with the question “Your relative income level in your local area.” Social status was measured with the question “Your social status level in your local area.” Responses of those 2 questions are rated on a 5-point Likert scale ranging from 1 (strongly low) to 5 (strongly high). Life satisfaction was measured with the question “Rating of your satisfaction with your life,” responses are rated on a 5-point Likert scale ranging from 1 (strongly dissatisfied) to 5 (strongly satisfied).

### 2.4. Grouping variables

Lifestyles (smoking, drinking, physical exercise) and health conditions (chronic disease, self-report health). The current smoking was measured with the question “Whether smoked cigarettes last month,” and the possible answers included the following 2 options: yes and no. The current drinking was measured with the question “Whether drank alcohol 3 times per week last month?,” and the possible answers included yes and no. Physical exercise was obtained by the question “Frequency of your physical exercise last month when not on vacation.” Chronic disease was obtained by the question “Whether had doctor-diagnosed chronic disease in past 6 months.” Self-rated health was reported by responders themselves on a 5-point Likert scale ranging from 1 (excellent) to 5 (poor).

### 2.5. Statistical analyses

LCA was performed to classify participants into homogenous classes or groups based on a set of observed variables. The Akaike Information Criterion, Bayesian Information Criterion (BIC) and BIC using sample size adjustment were applied to compare models with different numbers of classes. Lower values of these fit indices were deemed better model fit. The Lo–Mendell–Rubin-Adjusted likelihood ratio test and the bootstrapped likelihood ratio test were employed to compare models with adjacent numbers of classes.^[19]^ LCA analyses were performed using Mplus version 8.0. The quantitative variables were described using means (median) and standard deviations (quartiles). Categorical variables were described using counts and proportions. Differences in demographic characteristics between choice for TCM treatment or not were examined using the Student *t* test for continuous variables and the chi-square test for categorical variables. The binary logistic regression was conducted to examine the association between lifestyles and health conditions for choice of TCM doctors. The results of the regression models are summarized via odds ratios (ORs) and their 95% confidence intervals (CIs). Analysis was performed using Statistical Package for the Social Sciences (SPSS) version 24.0 (SPSS Inc., Chicago, IL).

## 3. Results

### 3.1. LCA: clustering of lifestyles and health conditions

A total of 24,173 subjects were included in this study. There were 5 kinds (exercise, smoking, alcohol use, chronic disease, and self-report health) of lifestyles and health conditions; models with 1 to 4 latent classes were estimated using LCA. Model-fit indices were presented in Table 1. As the number of estimated classes increased, the Akaike Information Criterion, BIC, and aBIC generally decreased, and entropy reached 0.80 in the 3-cluster model only, which indicated that the 3-cluster model was the target number of latent clusters.

**Table 1:** Model-fit indices for latent class factor analysis for lifestyle and health condition (N = 24,173).

	Npar*	LL^2^†	AIC‡	BIC§	aBIC∥	Entropy
1-cluster model	8	−85308.91	170633.81	170698.56	170673.13	–
2-cluster model	17	−83427.55	166889.11	167026.70	166972.67	0.62
3-cluster model	26	−82289.42	164630.85	164841.26	164758.64	0.80
4-cluster model	35	−82224.88	164519.77	164803.03	164691.80	0.72

* Number of parameters in the model.

† Log likelihood.

‡ Akaike information criterion.

§ Bayesian information criterion, based on the log likelihood.

∥ Bayesian information criterion using sample size adjustment.

**Figure 1.**
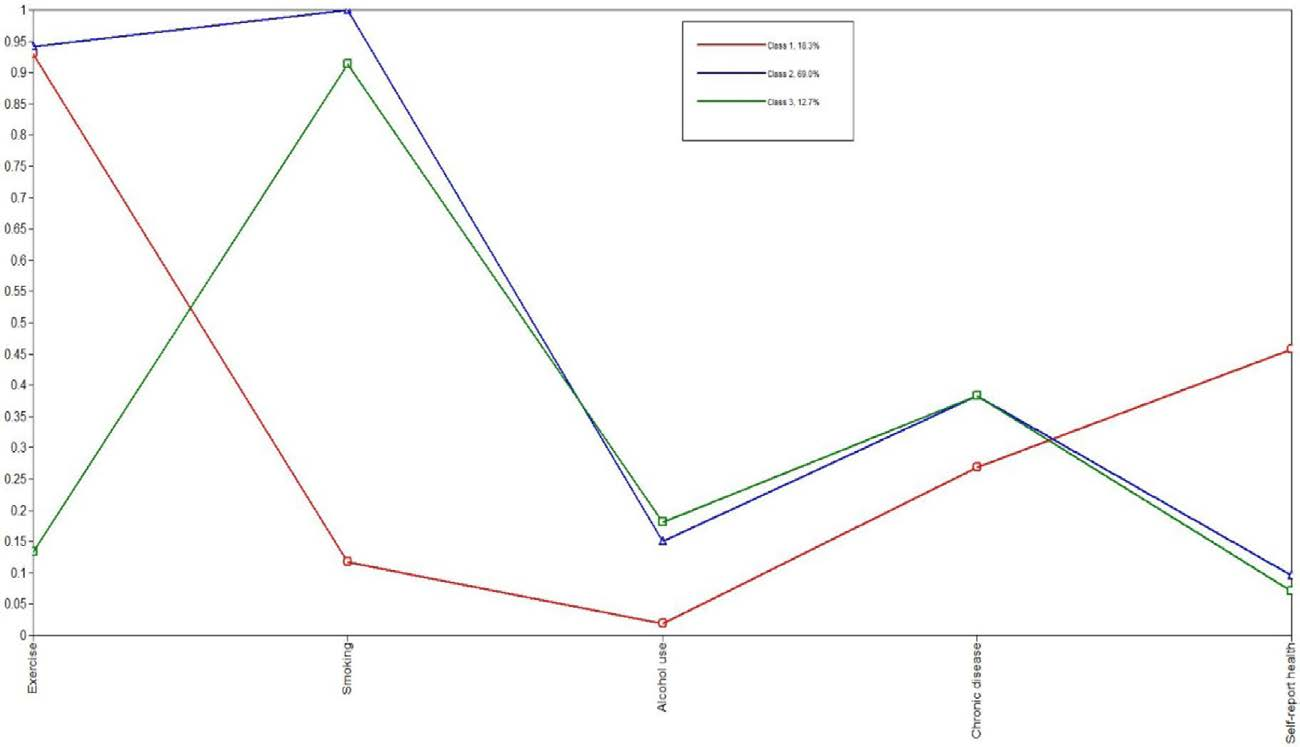
Cluster-specific probabilities of lifestyles and health conditions for the 3-cluster model.

Figure 1 exhibited the cluster-specific estimated probabilities of lifestyles and health conditions for the 3-cluster model from LCA. Class 1 accounted for 18.3% of the participants with a healthy pattern relative to the other 2 classes and was labeled the “healthy group.” Individuals in this class had relatively high probabilities of participating in physical exercise, with lower smoking and drinking, low probabilities of chronic disease and relatively high probabilities of self-report health. Class 2 accounted for 69.0% of the sample and can be characterized by their high probability of reporting unhealthy lifestyles, such as high probabilities of smoking and drinking, slightly high probability of chronic disease and low self-report disease but a high probability of exercise. As such, class 2 was labeled the “unhealthy behavior group.” Class 3 represented 12.7% of the sample and was distinguished from other classes by the highest probability of low physical exercise and was labeled the “physical inactivity group” (Fig 1.).

### 3.2. Demographic characteristics of participants

As displayed in Table 2, the average age of participants was 47.3 (standard deviation = 15.5) years, with a range from 16 to 95 years. Nearly half of the participants were female (49.3%), and were rural residents (51.4%). 27.3% of participants were illiterate, and most participants (83.3%) were married. More than half (57.5%) of individuals chose a TCM doctor. Participants who chose TCM treatment were more likely to be female, living in rural areas, with higher social status.

**Table 2:** Demographic characteristics.

	Total	Choice for TCM treatment		*t*/chi	*P* value
	(N = 24173)	Yes (N = 13891)	No. (N = 10282) *		
Age, mean (SD), yr	47.3 (15.5)	47.6 (15.1)	46.9 (16.1)	−3.27	.001
Gender, female, n (%)	11916 (49.3)	7023 (50.6)	4893 (47.6)	20.85	<.001
Education, n (%)					
None	6600 (27.3)	3857 (27.8)	2743 (26.7)	13.61	.003
Primary school	5501 (22.8)	3090 (22.2)	2411 (23.4)		
Junior high school	6958 (28.8)	3930 (28.3)	3028 (29.4)		
>Senior high school	5114 (21.2)	3014 (21.7)	2100 (20.4)		
Marital status, married, n (%)	20148 (83.3)	11669 (84.0)	8479 (82.5)		
Residency, rural, n (%)	12437 (51.4)	7484 (53.9)	4953 (48.2)	10.09	.001
Ever worked, yes, n (%)	17303 (71.6)	10102 (72.7)	7201 (70.0)	20.99	<.001
Social status, high, n (%)	4294 (17.8)	2568 (18.5)	1726 (16.8)	11.69	.001
Life satisfaction, strong, n (%)	654 (2.7)	371 (2.7)	283 (2.8)	6.82	.146
Lifestyle and health condition					
Healthy	3956 (16.4)	2527 (18.2)	1429 (13.9)	90.21	<.001
Unhealthy behavior	17733 (73.4)	10043 (72.3)	7690 (74.8)		
Physical inactivity	2484 (10.3)	1321 (9.5)	1163 (11.3)		

SD = standard deviation, TCM = traditional Chinese medicine.

* Compared with not choose TCM treatment.

### 3.3. The binary logistic regression model

As shown in Table 3, who were in unhealthy behavior group (smoking and drinking and with chronic disease) had significantly higher odds (OR = 1.51, 95% CI: 1.35–1.68, *P* < .001) to visit TCM doctors. At the same time, participants with less exercise (OR = 1.11, 95% CI: 1.02–1.22, *P* = .019) were positively associated with TCM treatment choice. When stratified by gender, this relationship still existed among males.

**Table 3:** Binary logistic regression between lifestyle and health condition and the choice of TCM.

Variables	Total (n = 24,173)		Male (n = 12,257)		Female (n = 11,916)	
	*P* value	OR (95% CI)	*P* value	OR (95% CI)	*P* value	OR (95% CI)
Age	.082	1.00 (1.00, 1.00)	.434	1.00 (1.00, 1.00)	.105	1.00 (0.99,1.01)
Education	<.001	1.07 (1.05, 1.10)	.052	1.04 (1.00, 1.08)	<.001	1.11 (1.05, 1.15)
Marital status, married	<.001	1.07 (1.04, 1.11)	.001	1.10 (1.04, 1.17)	.027	1.05 (1.01, 1.11)
Residency, rural	<.001	0.77 (0.73, 0.81)	<.001	0.73 (0.68, 0.79)	<.001	0.81 (0.74, 0.86)
Ever worked	<.001	1.16 (1.09, 1.23)	<.001	1.20 (1.10, 1.31)	.002	1.13 (1.04, 1.22)
Income level	.464	0.96 (0.88, 1.06)	1.861	1.01 (0.89, 1.15)	.170	0.90 (0.78, 1.04)
Social status	.015	1.09 (1.02, 1.17)	.132	1.08 (0.98, 1.20)	.124	1.08 (0.98, 1.20)
Life sanctification	.130	1.02 (0.99, 1.05)	.136	1.03 (0.99, 1.07)	.730	1.01 (0.97, 1.04)
Lifestyle and health condition (reference = healthy)						
Unhealthy behavior	<.001	1.51 (1.35, 1.68)	<.001	1.51 (1.33, 1.73)	.177	1.54 (0.82, 2.86)
Physical inactivity	.019	1.11 (1.02, 1.22)	.012	1.13 (1.03, 1.23)	.715	1.12 (0.60, 2.08)

CI = confident interval, OR = odds ratio, SE = standard error, TCM = traditional Chinese medicine.

* After controlling for age, gender, education, marital status, residency, ever worked, income level, social status and life sanctification.

## 4. Discussion

There is a growing trend toward traditional medicine, and traditional medicine exists in the world as a major source of health care, wellness, and preventive purposes for the majority of the population.^[7]^ To our knowledge, this is one of the first studies to identify associations between lifestyles, health conditions and TCM treatment choice among a large sample of the Chinese population using LCA with nationally representative data. Our results manifested there were that 57.7% of subjects chose TCM doctors. In this study, we found that individuals in the unhealthy behavior group and physical inactivity group had a higher value for receiving TCM treatment than others. This may be that those with bad health behaviors have a poor health condition that need recuperation, and TCM may be chosen when patients perceive the need for health maintenance or tonic care.^[20]^ Our results were consistent with a previous study; Chung et al reported individuals with drinking habits were preferred for TCM treatment.^[21]^ In addition, the possible reasons may be that subjects with chronic disease need to take Western medicine for a long time and which may cause significant side effects, whereas TCM is more thorough in “curing the root of the problem” even if it is considered to be slow in action.^[22]^ Those were consistent with several previous studies.^[16,23]^

Many researchers have reported that demographic variables^[24,25]^ and health factors^[26]^ were significant factors influencing the use of traditional and complementary medicine. The current study found a significant difference when TCM treatment choice was compared between age and education levels. On the one hand, this may be because old people have a greater sense of cultural identity and are more able to accept traditional medicine. On the other hand, the relatively old participants in subhealth states or with different levels of the chronic disease need more rehabilitation and health care interventions, so Chinese medicine is a suitable choice.^[27]^ The present study also found that individuals with high education was higher than that of people with low education; however, this was consistent with the previous study, which reported American women who have more education were more likely to use complementary medicine,^[28]^ but was inconsistent with the results reported by Liao and her colleagues.^[16]^ The possible reason may be that as individuals’ education level increases, their knowledge and understanding of Chinese medicine may be higher,^[29]^ and individuals with a higher education level with better financial conditions would pay more attention to their own health and with stronger needs for health preservation.^[27]^ Previous studies have shown that income level is a paramount factor for individuals seeking alternative care, complementary medicine users tend to have middle to high incomes.^[30,31]^ However, the influence of personal income level on TCM treatment choice was not statistically significant despite this being similar to another study’s results.^[16]^ One possible reason that the influence of income level on TCM treatment choice should be taken into consideration is not only personal income but also the total family income. In addition, a positive association between social status and TCM treatment choice was found under the multivariate analysis. Individuals with higher social status may have more available resources to access TCM treatment. Some American researchers have also indicated that complementary medicine users have a higher frequency of outpatient visits compared with nonusers.^[32]^

Given the increasing attention to TCM, the strength of the present findings has relevance for using a large sample of data to examine the influencing factors for the choice of TCM treatment, which may stimulate further research. Several limitations existed in the present study. First, this was an observational analysis, and causal inferences were not possible. Second, part information was collected based on self-reporting, which may involve information bias despite it being commonly used in the epidemiological study due to the feasibility consideration. Third, the sample is from investigations in China, so the findings may not be generalizable to other countries and regions.

## 5. Conclusions

The current study revealed the relationship between lifestyles, health conditions and the choice of TCM treatment. Individuals in unhealthy behavior or physical inactivity groups were more likely to visit TCM doctors than those in healthy groups. This will provide evidence for TCM development and provide support for further research.

## Author contributions

**Conceptualization:** Ning Yan.

**Data curation:** Xueping Ma, Mohan Wang, Juan Ma, Zhengjun Zhang.

**Design:** Ning Yan.

**Formal analysis:** Xueping Ma, Mohan Wang.

**Methodology:** Ning Yan.

**Supervision:** Yu Hao.

**Writing – original draft:** Xueping Ma, Mohan Wang.

**Writing – review & editing:** Ning Yan.
